# Whole genome sequence data of *Comamonas sediminis* FS4_11, a fumonisin B1-transforming bacterium, using hybrid nanopore-illumina sequencing

**DOI:** 10.1016/j.dib.2025.111829

**Published:** 2025-06-24

**Authors:** Yang Wang, Mengru Zhao, Zhe Wang, Xiaohong Luo, Chengfei Wang, Baoyuan Guo

**Affiliations:** a Institute of Grain and Oil Quality and Safety, Academy of National Food and Strategic Reserves Administration*,* Beijing 100037*,* China; b School of Health Science and Engineering, University of Shanghai for Science and Technology*,* Shanghai 200093*,* China; c Jiangsu Aomai Bio-Technology Co., Ltd.*,* Nanjing 211226*,* China

**Keywords:** Comamonas sediminis, Mycotoxin, Hybrid sequencing, Genome assembly, Functional annotation, Biosafety

## Abstract

The genome of Comamonas sediminis FS4_11, a bacterial strain with mycotoxin fumonisin B1 (FB1) transformation capability, was sequenced using Oxford Nanopore Technologies (ONT) and Illumina platforms. The final assembly generated a circular chromosome of 5,148,490 bp with a mean G+C content of 63.74%, representing a contiguous genomic structure. Genome annotation predicted 4565 protein-coding sequences (CDSs), 82 transfer RNAs (tRNAs), 18 ribosomal RNAs (rRNAs; 6 each of 5S, 16S, and 23S rRNA), 1 transfer-messenger RNA (tmRNA), and 8 pseudogenes and other non-coding RNAs. Functional annotation identified 939 potential virulence factors, two putative AdeF-related antibiotic resistance genes, 1486 potential pathogen-host interaction proteins, and a candidate carboxylesterase for FB1 transformation. This dataset primarily aids in identifying potential FB1 detoxification enzyme genes and assessing strain biosafety. It also offers significant reuse potential for comparative genomics and understanding bacterial evolution.

Specifications Table

**Table:** 

Subject	*Microbiology: Applied Microbiology*
Specific subject area	*Microbiology, Genomics, Biotechnology*
Type of data	Tables, Figures
Data collection	High-quality genomic DNA was extracted using the Wizard® genomic DNA purification kit (Promega). Sequencing utilized Oxford Nanopore PromethION 48 for long reads and Illumina NovaSeq 6000 for short reads. Nanopore reads were filtered with Filtlong v0.2.0. Hybrid assembly combined Canu v1.5 (ONT draft), Circlator v1.5.5 (circularization), Racon v1.4.3 (polishing), and Pilon v1.22 (Illumina error correction). Gene prediction used Bakta (software v1.11 and database v6.0.0). Predicted proteins were further analyzed against specialized databases for antibiotic resistance (CARD v3.2.7), pathogen-host interactions (PHI-base v4.17), and virulence factors (VFDB 2022).
Data source location	Institution: Academy of National Food and Strategic Reserves Administration City: Beijing Country: China
Data accessibility	Repository name: National Centre for Biotechnology Information (NCBI) Data identification number: BioProject Accession Number: PRJNA1258737, BioSample: SAMN48330266, NCBI SRA Accession Number: SRR33422104 and SRR33422105, NCBI GenBank Accession Number: CP191350 https://www.ncbi.nlm.nih.gov/bioproject/PRJNA1258737 https://www.ncbi.nlm.nih.gov/biosample/?term=SAMN48330266 https://www.ncbi.nlm.nih.gov/sra/SRX28663058 https://www.ncbi.nlm.nih.gov/sra/SRX28663057 https://www.ncbi.nlm.nih.gov/nuccore/CP191350
Related research article	N/A

## 1 Value of the Data

• This genome of *C. sediminis* FS4_11 provides the essential foundation for identifying and characterizing genes potentially responsible for fumonisin B1 transformation. Researchers can use this sequence data as a reference to search for candidate genes based on homology to known FB1-degrading enzymes.
• The genome serves as a valuable reference for comparative genomics studies, contributing to our understanding of bacterial evolution and diversity within this species group.
• The comprehensive annotation of virulence factors, antibiotic resistance genes, and pathogenicity-related elements offers essential data for biosafety assessment, supporting researchers in evaluating the strain's safety for industrial applications.

## 2 Background

Fumonisin B1 (FB1) is a major mycotoxin that poses significant risks to food safety and human health [1]. While microbial degradation offers a promising approach for FB1 detoxification, the molecular mechanisms remain poorly understood [2]. *C. sediminis* FS4_11, isolated from a maize frass composite from *Sitophilus zeamais* infestation, exhibited FB1 biotransformation capability. Similar activity has been reported in the phylogenetically closely related strain NCB 1492 (*Delftia*/*Comamonas*-like), though the enzymes responsible remain unidentified in the *Comamonas* genus [3]. To investigate the genetic basis of this biotransformation and evaluate strain biosafety, we performed whole-genome sequencing using a hybrid approach combining Oxford Nanopore long-read and Illumina short-read technologies. The genome data provides a bioinformatic foundation for identifying potential FB1-degrading enzyme genes and enables biosafety assessment through detection of virulence factors, antibiotic resistance genes, and other safety-related genetic elements.

## 3 Data Description

**Fig. 1:**
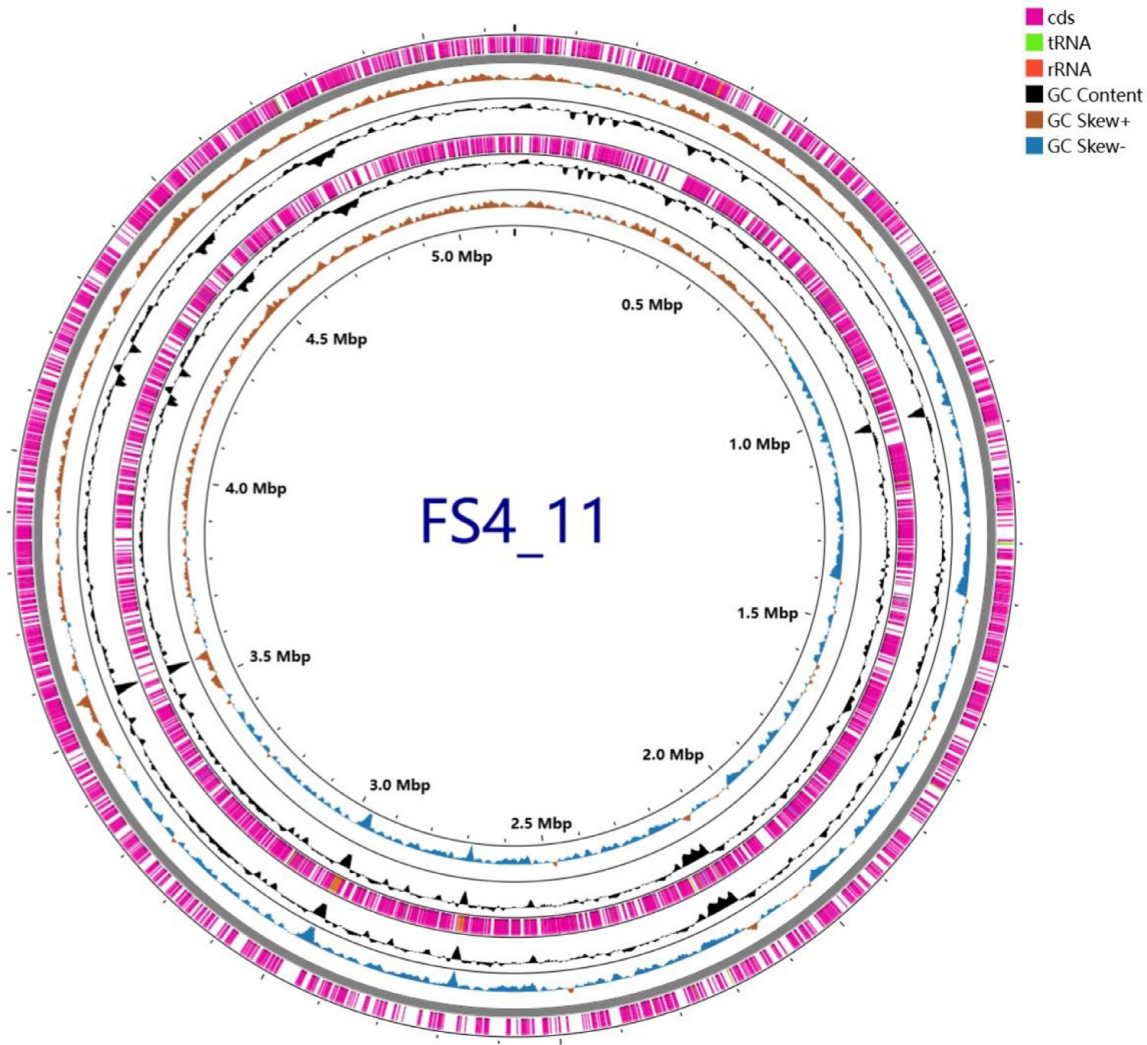
Circular genome map of *C. sediminis* FS4_11 generated using Proksee. The map displays genomic features arranged in concentric circles. From the outermost circle inwards: (1 & 2) Predicted coding sequences (CDS) on the forward (sense) and reverse (antisense) strands (magenta). (3) Transfer RNA (tRNA, green) and ribosomal RNA (rRNA, orange) genes. (4) GC content variation plot (black, deviation from the mean of 63.74%). (5) GC skew ((G-C)/(G+C)), with positive values shown in orange/brown and negative values in blue. The scale markers indicate the genome size in megabase pairs (Mbp), corresponding to the total genome size of 5,148,490 bp.

The genomic statistics for strain FS4_11 are summarized in Table 1. The genome consists of a single contig representing a chromosome (5,148,490 bp; N50 5,148,490 bp) with a GC content of 63.74% and an average coverage depth of 399.5x. Annotation predicted 4,565 protein-coding sequences (CDS), 82 tRNA genes, 1 transfer-messenger RNA (tmRNA) gene, 8 pseudogenes, and 18 rRNA genes (6 copies each of 5S, 16S, and 23S). Additionally, 8 non-coding RNA (ncRNA) genes and 18 ncRNA regions were identified. No clustered regularly interspaced short palindromic repeats (CRISPR) was found. The overall genome structure and distribution of these features are visualized in Fig. 1.

**Table 1:** Genome statistics and features of strain FS4 11.

Feature	Value
Genome Length (bp)	5,148,490
GC content (%)	63.74
Number of contigs	1
tRNA	82
tmRNA (Transfer-messenger RNA)	1
rRNA (5S, 16S, 23S)	6, 6, 6
ncRNAs	8
ncRNA region	16
CDSs	4,565
Pseudogenes	8
CRISPR	0
N50 value	5,148,490
Genome Coverage (×)	399.5
Completeness	91.73%
Contamination value	5.28%

Comparison of predicted protein sequences against the Virulence Factor Database (VFDB) via BLASTp (E-value < 1e^-5^) yielded 939 potential homologs to known virulence factors. These contained various virulence-associated functions, including adhesion, toxin production (e.g., Colibactin, Cytolysin, and Hemolysin), secretion systems (e.g., T3SS, T4SS, and T7SS), immune modulation, surface structures (e.g., O antigen and Vi antigen), and multiple iron uptake pathways (e.g., Pyoverdine, Pyochelin, Aerobactin, Yersiniabactin, FeoAB, and Legiobactin).

Comparison of the predicted protein sequences against the PHI-base database identified 1486 potential pathogen-host interaction (PHI) related proteins. Functional analysis of these candidates revealed homologs associated with a diverse range of processes potentially involved in host interactions, including factors related to iron acquisition (e.g., homologs of ferrichrome receptor, proteins related to Fe3+ transport, Fec operon regulator, and enterobactin synthetase component A), potential plant hormone manipulation (proteins possibly associated with auxin biosynthetic pathways), host tissue degradation (e.g., putative collagenase), metabolism and stress response (e.g., cystathionine beta-lyase and terminal oxidases like cytochrome O ubiquinol oxidase subunit CyoA), transport and efflux systems (e.g., ABC-type efflux pumps), and regulatory functions (e.g., ECF sigma factors).

To identify the gene responsible for FB1 transformation in strain FS4_11, a homology search was initially performed using the characterized FB1 hydrolase from *Sphingopyxis macrogoltabida* (UniProtKB D2D3B6.1) [4] as a query. However, this BLASTp analysis (E-value cutoff < 1e^-5^) against the predicted proteome of FS4_11 did not yield any significant homologs. Consequently, we turned to functional annotations to identify potential candidates. Examination of the high-quality, manually curated Swiss-Prot database annotations for FS4_11 revealed a single gene annotated as a carboxylesterase. As this enzyme belongs to the type-B carboxylesterase/lipase family, consistent with the family of the known FB1 hydrolase used as the initial probe, this gene was selected as the primary candidate for encoding the FB1-hydrolyzing activity in strain FS4_11.

**Fig. 2:**
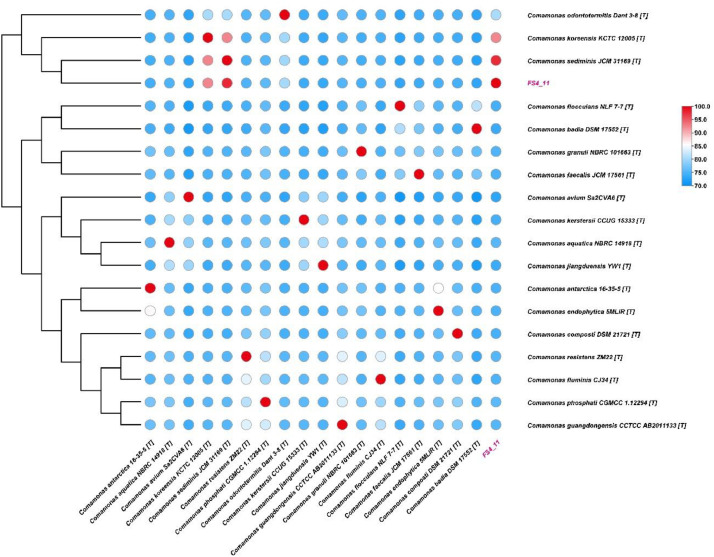
Heatmap of pairwise ANIb values comparing strain FS4_11 to *Comamonas* type strains.

The pairwise Average Nucleotide Identity based on BLAST (ANIb) values between FS4_11 and related type strains are visualized in the heatmap presented in Fig. 2. The colour gradient, ranging from blue (lower identity) to red (higher identity), clearly delineates genomic relatedness, which is further supported by the hierarchical clustering shown in the accompanying dendrogram. Consistent with the quantitative results, the heatmap visually confirms the highest genomic similarity between strain FS4_11 and *C. sediminis* JCM 31169^T^ (indicated by the deep red colour corresponding to 97.65% ANIb). Furthermore, FS4_11 clusters closely with *C. koreensis* KCTC 12005^T^and *C. odontotermitis* Dant 3-8^T^, showing relatively high ANIb values with these strains and forming a distinct clade. Overall, the heatmap analysis corroborates the phylogenetic placement of FS4_11 within the *Comamonas* genus, highlighting its specific affiliation with *C. sediminis* and closely related species.

Screening against the CARD database identified two potential antibiotic resistance genes (ARGs). Both were identified via the protein homolog model as encoding homologs related to AdeF, a component of resistance-nodulation-cell division (RND) family antibiotic efflux pumps. These two AdeF homologs exhibited sequence identities of 71.36% and 58.54%, respectively, to known resistance determinants in CARD. Based on their homology, these efflux pumps are predicted to contribute to resistance primarily against fluoroquinolone and tetracycline antibiotics.

## 4 Experimental Design, Materials and Methods

### 4.1 Strain Source and Culture Conditions

*S*train FS4_11, known for its FB1 degradation activity, was isolated from maize frass associated with *Sitophilus zeamais* infestation as part of a previous screening effort by our lab. The detailed methodology for isolating strain FS4_11 and confirming its FB1 degradation potential will be reported in a future publication. For DNA extraction, a fresh single colony grown on Nutrient Agar (NA) was picked and inoculated into 5 ml of Nutrient Broth (NB). The culture was incubated at 30°C with shaking at 180 rpm for 18 h. Bacterial cells were subsequently harvested by centrifugation at 12,000 x g for 10 min at 4°C.

### 4.2 DNA Extraction and Quality Assessment

Genomic DNA was extracted using the Wizard® Genomic DNA Purification Kit (Promega) following the manufacturer’s protocol. The genomic DNA quality was assessed using a NanoDrop 2000 (Thermo Fisher Scientific) for purity, a Qubit 4 Fluorometer (Thermo Fisher Scientific) for concentration, and agarose gel electrophoresis for integrity.

### 4.3 Genome Sequencing, Assembly, Quality Assessment and Annotation

Genome sequencing was outsourced to Biomarker Technologies (Beijing, China) using a hybrid approach combining long-read (Oxford Nanopore Technologies, ONT) and short-read (Illumina) sequencing. For long-read sequencing, genomic DNA library was prepared using the SQK-LSK109 ligation sequencing kit and processed on a PromethION 48 instrument (ONT, Oxford, UK) using a PromethION R9.4.1 flow cell. Raw Nanopore reads were filtered using Filtlong v0.2.0 (https://github.com/rrwick/Filtlong) to remove low-quality reads (mean quality score < 6) and/or short reads (length < 2,000 bp). The filtered reads were assembled using Canu v1.5 [5], and the resulting assembly was then circularized using Circlator v1.5.5 [6]. Subsequently, this circularized assembly was polished using the filtered Nanopore reads with Racon v1.4.3 [7]. For short-read sequencing, a paired-end library with an approximate insert size of 350 bp was constructed using the Hieff NGS® OnePot Pro DNA Library Prep Kit for Illumina (Yeasen Biotechnology Co., Ltd., Shanghai, China). This library was sequenced on an Illumina NovaSeq 6000 platform (Illumina, USA) to generate 2 × 150 bp paired-end reads. The quality of the raw Illumina reads was assessed using FastQC v0.11.5 (https://www.bioinformatics.babraham.ac.uk/projects/fastqc/). These reads were then mapped to the Racon-polished assembly using Burrows-Wheeler Aligner (BWA-MEM) v0.7.19 (https://github.com/lh3/bwa). Finally, the mapped Illumina reads were used to perform further sequence and assembly error correction using Pilon v1.23 (https://github.com/broadinstitute/pilon/releases/) to obtain the final polished genome sequence. The assembled genome was visualized as a circular map using the CGView web service (https://proksee.ca/) [8]. Genome completeness and contamination were assessed using CheckM v1.2.3 (https://github.com/Ecogenomics/CheckM?tab=readme-ov-file). All software programs were executed using their default parameters unless otherwise specified.

Structural and functional annotation of the final polished genome was performed. Coding sequences (CDS), transfer RNA (tRNA) genes, and ribosomal RNA (rRNA) were predicted using Bakta web (https://bakta.computational.bio/, software v1.11.0 and database v6.0.0) [9]. Predicted proteins were functionally annotated by BLASTp comparison against the Swiss-Prot database [10] (E-value < 1e^-5^). Further functional insights were gained by searching protein sequences against specialized databases relevant to antibiotic resistance (CARD; Comprehensive Antibiotic Resistance Database v3.2.7 [11]), pathogen-host interactions (PHI; Pathogen-Host Interaction database v4.17 [12]), and virulence factors (VFDB; Virulence Factor Database 2022 [13]).

### 4.4 Average Nucleotide Identity (ANI) Analysis

To assess the genomic relatedness of *C. sediminis* FS4_11 to other species within the genus, Average Nucleotide Identity based on BLAST (ANIb) was calculated using the JSpeciesWS web service [14]. The genome sequence of FS4_11 was compared against the genome sequences of selected *Comamonas* type strains. Default parameters were used for the ANIb calculation. The resulting pairwise ANIb values were visualized as a heatmap using TBtools v1.098769 [15].

## Limitations

Not applicable.

## Ethics Statement

The current work does not involve human subjects, animal experiments, or any data collected from social media platforms.

## CRediT authorship contribution statement

**Yang Wang:** Conceptualization, Methodology, Investigation, Formal analysis, Writing – original draft, Funding acquisition. **Mengru Zhao:** Resources, Investigation, Writing – review & editing. **Zhe Wang:** Validation, Investigation, Data curation, Visualization. **Xiaohong Luo:** Investigation, Writing – review & editing. **Chengfei Wang:** Writing – review & editing. **Baoyuan Guo:** Conceptualization, Supervision, Project administration.

## Data Availability

ncbiBioProject (Original data)
NCBISRA data_ONT (Original data)
NCBISRA data_Illumina (Original data)
NCBIgenome (Original data)
ncbiBioSample (Original data)
